# FSPP: A Tool for Genome-Wide Prediction of smORF-Encoded Peptides and Their Functions

**DOI:** 10.3389/fgene.2018.00096

**Published:** 2018-04-05

**Authors:** Hui Li, Li Xiao, Lili Zhang, Jiarui Wu, Bin Wei, Ninghui Sun, Yi Zhao

**Affiliations:** ^1^Key Laboratory of Intelligent Information Processing, Advanced Computer Research Center, Institute of Computing Technology, Chinese Academy of Sciences, Beijing, China; ^2^School of Computer and Control Engineering, University of Chinese Academy of Sciences (UCAS), Beijing, China; ^3^CAS Key Laboratory of RNA Biology, Institute of Biophysics, Chinese Academy of Sciences, Beijing, China; ^4^Department of Clinical Pharmacology of Traditional Chinese Medicine, School of Chinese Materia Medica, Beijing University of Chinese Medicine, Beijing, China; ^5^State Key Laboratory of Computer Architecture, Institute of Computing Technology, Chinese Academy of Sciences, Beijing, China

**Keywords:** smORF, SEP, Ribo-seq, MS, function

## Abstract

smORFs are small open reading frames of less than 100 codons. Recent low throughput experiments showed a lot of smORF-encoded peptides (SEPs) played crucial rule in processes such as regulation of transcription or translation, transportation through membranes and the antimicrobial activity. In order to gather more functional SEPs, it is necessary to have access to genome-wide prediction tools to give profound directions for low throughput experiments. In this study, we put forward a functional smORF-encoded peptides predictor (FSPP) which tended to predict authentic SEPs and their functions in a high throughput method. FSPP used the overlap of detected SEPs from Ribo-seq and mass spectrometry as target objects. With the expression data on transcription and translation levels, FSPP built two co-expression networks. Combing co-location relations, FSPP constructed a compound network and then annotated SEPs with functions of adjacent nodes. Tested on 38 sequenced samples of 5 human cell lines, FSPP successfully predicted 856 out of 960 annotated proteins. Interestingly, FSPP also highlighted 568 functional SEPs from these samples. After comparison, the roles predicted by FSPP were consistent with known functions. These results suggest that FSPP is a reliable tool for the identification of functional small peptides. FSPP source code can be acquired at https://www.bioinfo.org/FSPP.

## Introduction

There has been an arbitrary cut-offs in metazoans that genes are totally divided into protein-coding (messenger RNA referred to as mRNA) and non-coding sequences (Doerks et al., 2002; Kapranov et al., 2002; Bertone et al., 2004; Carninci et al., 2005). mRNA sequences carry open reading frames (ORF) which can be translated into polypeptides composed of more than 100 codons (Niehrs and Pollet, 1999). Then polypeptides will fold into distinct structural units (domains) and acquire specific functions. On the other hand, non-coding RNAs cannot be translated into proteins but are involved in many cellular processes (Wadler and Vanderpool, 2007; Dinger et al., 2008; Ender et al., 2008).

A more complete understanding of the molecular complexity of genes has been recently demonstrated (Ruiz-Orera et al., 2014). The emerging ribosome profiling technology uncovered thousands of smORFs with the length smaller than 100 codons (Basrai et al., 1997) are being translated (Bazzini et al., 2014; Smith et al., 2014). While the advanced technologies provided additional evidence of smORF-encoded peptides (SEPs) which used to be considered useless and were deemed non-coding because they lack propensity to form known protein domains. Recently, CRISPR–Cas-based gene editing tools have elucidated the targeted manipulation of individual SEPs and herald a promising new era for SEPs functions (Couso and Patraquim, 2017). The results demonstrated that SEPs encoded by smORFs were fulfilling key physiological functions (Anderson et al., 2015; Nelson et al., 2016).

smORF-encoded peptides function through multiple sorts of mechanisms. First, these small peptides, especially those encoded by upstream ORFs (uORFs), are closely related with the translation of canonical mRNAs. The generations of SEPs interfere with the translation of their associated downstream proteins by regulating the passage of ribosomal subunits on a 5′ leader sequence (Andrews and Rothnagel, 2014). Second, SEPs cannot support the typical, multi-domain structure of canonical proteins but instead accommodate only one or, at most, two simple protein domains. The dominant-negative feature specifically fits the small size of SEPs. SEPs interfere with the function of canonical transcription factors, either by sequestering them into unproductive dimers or by competing with them for binding to DNA (Couso and Patraquim, 2017). Third, SEPs have an increased frequency of some positively charged amino acids, thereby producing an overall positive charge bias. This positive charge feature similarly favors their interactions with the negatively charged mitochondria and supplies the SEPs with the property to act as the positively charged cell-penetrating peptides by crossing cellular membranes and organelles. Forth, the cationic amino acid of SEPs exhibit similar mode of action as antimicrobial peptides. Antimicrobial peptides are characterized by heir propensity to form αTMHs and are also referred to as amphipathic peptides (Fan et al., 2016). This amphipathic property confers solubility and the ability to bind to and integrate into microbial membranes (Wenzel et al., 2014).

Overall, SEPs have an active role in various biological processes independently or by binding to canonical proteins or other cellular factors. Problems have occurred during the study of functional SEPs with regards to poor experimental and bioinformatics limitations. Some of the greatest challenging research in this field would be to overcome these limitations and to increase the pool of experimental conditions of SEPs. CRISPR/Cas9 technology has emerged as the most popular tool for genome engineering. This system enables editing of individual SEPs and has the potential to study the function with efficiency (Liu et al., 2017; Zhang et al., 2017). However, CRISPR system is a low DNA input method and relies on studying the roles of individual SEPs. There is an urgent need to develop genome-wide techniques to direct the low-throughout experiments. Some studies such as SmProt (Hao et al., 2017) used InterProScant (Jones et al., 2014) to predict the function through the protein domains identifier. As mentioned above, SEPs cannot support the typical, multi-domain structure of canonical proteins but instead accommodate only one or, at most, two simple protein domains. So this domains identifier approach is fundamentally not proper for SEP function prediction.

In this study, we realized a novel tool named functional smORF-encoded peptides predictor (FSPP) which relies on network to widely predict functional SEPs. We suppose that SEPs’ regulations on other targets can be detected through the co-expression network. In this way, we predict the SEP function with the help of the targets. Network-based function prediction has been proposed for predicting protein function in early 2001 when Hishigaki annotated the protein with protein-protein interaction network (Hishigaki et al., 2001). Besides, ncFANs is the first web server that relies on calculating coding and non-coding gene expression networks to predict lncRNA functions (Liao et al., 2011). Lnc-GFP was developed to predict non-coding RNA functions based on integrated gene expression and protein interaction data (Guo et al., 2013). In this paper, FSPP made use of the newly developed ribo-seq, mass spectrometry and RNA-seq technology to explore the functions of the new focus, small peptides. Compared with similar tools, FSPP is more suitable for SEPs due to two features. Firstly, it combines several advanced methods to acquire the most authentic SEPs. Secondly, it realizes a more specific approach for the prediction of small peptides functions.

## Materials and Methods

### Platform

**FIGURE 1:**
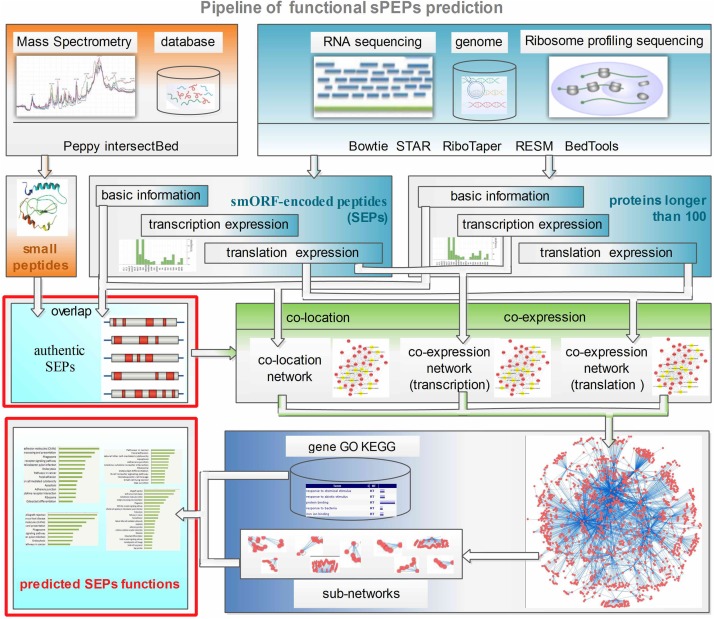
Pipeline of FSPP. Ribo-seq, RNA-seq and mass spectrometry data are input into FSPP. This tool integrates RiboTaper, Peppy etc. to analyze the three kinds of input and gets the fundamental and expression information of translated products. FSPP uses the overlap results of MS and Ribo-seq as the authentic target SEPs. Co-expression relevance and co-location information are used to build up the relation networks. FSPP selects the sub-networks which are significantly related with the target SEPs and annotate the SEPs’ functions with the help of the known neighbors. FSPP, functional smORF-encoded peptides predictor; MS, mass spectrometry; SEPs, smORF-encoded peptides.

As mentioned above, the mechanism of action of SEPs is to regulate the translation of other proteins or to interfere with transcription factors during the transcription process. Thus there is an inference that SEPs expression abundances should be closely correlated with those of target proteins and RNAs. So the functions of SEPs might be determined by the annotation of targets through the analysis of polypeptides and transcripts expression. Taking advantage of SEPs sample specific expression profile, FSPP built a relation network and functionally annotated SEPs by its neighbors in the network. The pipeline of functional SEPs prediction is shown in **Figure 1**.

### Identification of Authentic SEPs

FSPP designed a pipeline that used three kinds of data to provide predictions of functional SEPs. Due to the spatial and temporal expression features, some annotated SEPs may not be completely identified in the specific sequenced samples. Thus, to systematically study functional SEPs, it is first necessary to establish which sets of SEPs are authentic.

The identification of SEPs remains a great challenge owing to its small size and low abundance, leading to difficulties during the isolation and enrichment of peptides (Andrews and Rothnagel, 2014). Ribo-seq is a method used to globally investigate protein synthesis by deep sequencing RNA fragments protected by engaged translating ribosomes. Since its first description, Ribo-seq has been used to investigate small peptides (Calviello et al., 2016). However, several studies proposed that the mere presence of Ribo-seq reads in regions of the transcriptome did not imply the presence of actively elongating ribosomes (Guttman et al., 2013; Ingolia et al., 2014). Fortunately, the mass spectrometry provided a very different technology to detect translated products. Notably, each of the two methods has its own false-positive SEPs and their combination offers the possibility to identify the proper expressed SEPs. FSPP has integrated RiboTaper (Calviello et al., 2016) to process Ribo-seq and resulted in the translation production sets including SEPs and other proteins longer than 100. On another hand, Peppy (Risk et al., 2013) was used to match the MS spectra to the reference genome and obtained the best genomic location for each spectrum. At last, FSPP intersected the two results from Ribo-seq and mass spectrometry, and the overlap was considered as authentic SEPs set of the experiments.

With RNA-seq methods, FSPP acquired three kinds of information for the translated products: basic information, translation expression and transcription expression. The basic information includes products length, location in genome, transcript ID in Ensembl, peptide sequence and etc. FSPP uses ribosome density to measure the translation expression. Although the density varies across a transcript due to the different speed at different positions within a reading frame, independent lines of evidence suggest that the average occupancy across an entire gene corrects the consequences of this local variation (Ingolia et al., 2011). RSEM (Li and Dewey, 2011) is used to calculate the expression of proteins and SEPs at the transcription and translation level. The measurement of the proportion is TPM (transcripts per million) unit. The approach assumes that three or more samples of interest must be designed to enhance the quality of expression profiles.

### Prediction of SEPs’ Functions

As mentioned above, SEPs regulate other proteins during the two-step process of transcription and translation. Thus, SEPs expression should be closely related with those of target proteins and RNAs. In theory, the regulation targets can be acquired from the expression matrixes. FSPP constructed two expression matrixes. The first matrix is to identify SEPs functions in the translation process based on the SEP translation profile and the other proteins translation expression. The second is combined with SEP translation expression and the other proteins transcription profile to explore SEP regulation role in the transcription process. The statistically significant correlations are depicted from the matrixes. Accordingly, the chosen targets are labeled with the correlation coefficient value.

Correlation network is constructed according to the correlations among SEPs and larger proteins. Besides the connections mined from the two matrixes, FSPP network also includes the co-location relations between SEPs and proteins. This kind of relationship are defined that SEPs and the targets are from the same RNA. FSPP defines every related target as vertexes and the relations as edges. Therefore, this is the relation network in which FSPP carries out based-function prediction.

Based on the relation in the network, FSPP uses two different methods to predict functional peptides: module-based method and hub-based method. In the module-based method, FSPP performs the Markov clustering algorithm with default parameters to identify modules in the relation network. Based on the hypothesis the related items in a module often represent relevant functional units (e.g., molecular complexes or pathways), SEPs are then assigned functions that enriched among the coding genes in the same module. The second method consists of selecting the hubs by a user-defined cut-off for the node degree, and the functions that are enriched among its immediate coding gene neighbors will be assigned to the SEP. In the current version of FSPP, the functional annotations include Gene Ontology (Ashburner et al., 2000) biological process (BP) description and the statistical significance of the functional enrichment.

## Results

To test FSPP, we downloaded 38 human ribosome profiling data sets (RNA-seq) covering 5 cell lines from GEO database. The 38 sets included two HCT116 cell-line samples, five HEK293 cell-line samples, fifteen HEK293T cell-line samples, seven Hela cell-line samples and nine BJ cell-line samples (**Supplementary Table 1**). Similarly, the corresponding RNA-seq data sets were also downloaded. The respective MS data were obtained from EMBL-EBI PRIDE Archive (Vizcaino et al., 2016) and smPort (Hao et al., 2017).

### Network Statistics

In the step of identification, 83677 ORFs under translation were found from the Ribo-seq and RNA-seq data, 18268 of which were from short ORFs. In particular, 2663 of these smORF peptides overlapped with the results of MS data analysis (**Supplementary Table 2**).

**FIGURE 2:**
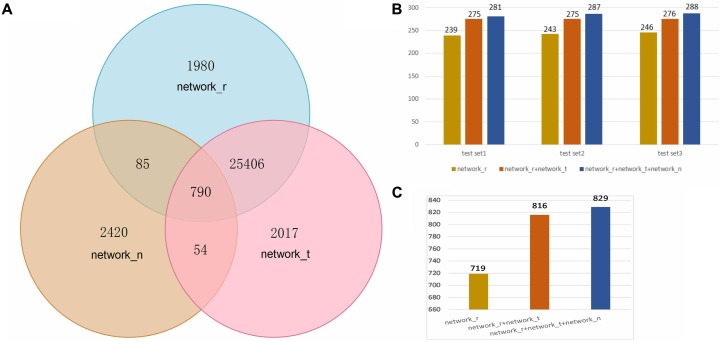
**(A)** Statistics of nodes in three networks. Network_r: co-expression relations of RNA level; network_t: co-expression relations of translation level; network_n: relations of neighbors. **(B)** Numbers of predicted peptides in three test sets from three networks. **(C)** Numbers of predicted peptides in the global test data.

The next step was to analyze 2663 SEPs expression profiles of 38 Ribo-seq samples in combination with the other 81915 protein transcription profiles, and this is presented as the first expression matrix. A Pearson’s correlation coefficient, r, measures the strength of the linear relationship between every two rows. 28261 significantly related pairs (network_r) passed through the filter criterion with an absolute pcc value larger than 0.97 and a *p-*value less than 0.01. In addition, 2663 SEPs translation expression data were mixed with 81915 protein translation profiles in 38 RNA-seq samples and formed the second expression matrix. After the filter step, 28267 significantly co-expressed items were selected. Combined with the 28261 items in the translation matrix, 30332 outstanding related nodes generated altogether a co-expression network. As for co-location relations, 3349 of SEPs have been found their pairs in the same RNAs. In total, 32752 nodes constructed the annotation network of FSPP. The node intersections among the three networks are illustrated in **Figure 2A**.

### Evaluation Using Known Proteins

To value FSPP results, 1% (320) of the annotated proteins was randomly selected from the 32752 network nodes. This step was repeated three times and listed as test set1, test set2 and test set3. All the GO annotations of these 960 proteins were eliminated and their functions were reproduced. At last, annotated proteins detected for the three sets are respectively 281, 287, and 288 (**Figure 2B**). All in all, 856 functional proteins were predicted by FSPP. The detectable rate of these 960 items was 89% (**Supplementary Tables 3**–**5**).

This evaluation was tested in three networks; RNA co-expression network (network_r), translation co-expression network (network_t) and co-location network (network_n). These networks represent three relations: RNA transcription regulation targets, translation regulation targets and co-location relation. As shown in **Figure 2A**, the number of RNA network_r nodes is consistent with that of network_t and are far more than network_n. It implied that much more functions can be found through the expression correlation than location relation. There are 25406 nodes overlap between network_r and network_t which is expected. At the same time 2420 nodes specially belong to network_n. It means the co-location relation is necessary in the network.

We define network_rt is the combination of network_r and network_t. Network_rtn is the total prediction network including network_r network_t and network_n. As shown in **Figures 2B,C**, the number of detected proteins in network_rt was significantly higher than those from network_r. In the other hand, proteins in network_rtn were slightly more than those of network_rt. For the total test data, 719 functional proteins were annotated from network_r. 816 were detected from network_rt. And 829 unique annotated proteins were from network_rtn.

### Comparisons of Hub and Module Methods

**FIGURE 3:**
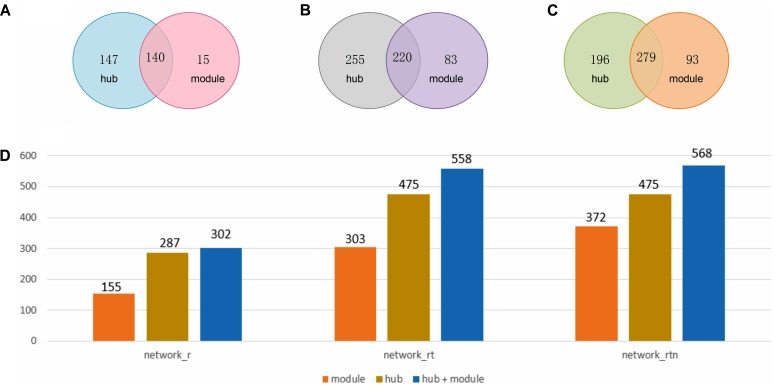
Statistics of SEPs hub and module methods in three networks. **(A)** The count of hub nodes and module nodes in RNA co-expression network (network_r). **(B)** The count of hub nodes and module nodes in RNA and translation products co-expression network (network_rt). **(C)** The count of hub nodes and module nodes in co-expression and neighbor relation network (network_rtn). **(D)** Box plot of nodes distribution of hub and module methods in three networks.

As mentioned above, FSPP adopted two methods, one based on hub and the second on modules, to detect the annotation sub-network of unknown peptides. After the optimization, the detected hubs were defined and singly treated as it contains one unknown SEP and at least five immediate neighbors with gene ontology biological process annotations (Lluch-Senar et al., 2015). At the same time, modules were chosen with an inflation parameter value of 1.8 and at least 15 nodes with known function in one module of MCL algorithm. **Figure 3** illustrates that peptides detected by hub methods were a little more than those detected by module methods in three networks. In total, 568 functional SEPs were identified by FSPP.

### Comparisons of Known Annotations

We compared the predicted functions with the reported SEPs’ functions. The results implied that FSPP is a reliable tool to predict SEP function. The three examples described in **Figure 2** illustrate it.

**FIGURE 4:**
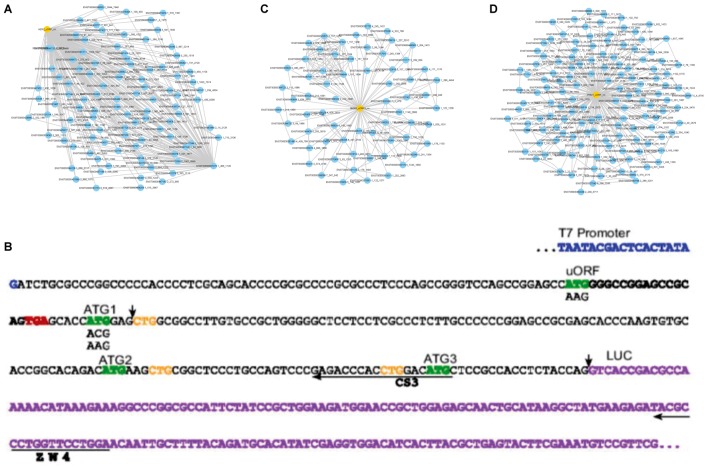
The detailed sub-networks to give functions. **(A)** Sub network to predict HER2_uORF functions. **(B)** The 5′ leader regions of HER2 sequence annotated by Sachs team (Spevak et al., 2006). FSPP-detected black bold sequence produced a 6-length functional peptide. **(C)** Sub-network to predict MKKS_uORF functions. **(D)** Sub-network to predict IFRD1_uORF functions.

In 2006, Matthew S. Sachs etc. revealed that synthesis of HER2 is controlled in part by an upstream open reading frame present in the transcript. The uORF reduced translation of the downstream protein in all systems and also affected downstream start-site selection. FSPP successfully detected the functional translation product from the uORF of HER2 (**Figure 4B**). The functions were mainly focused on measuring the effects on downstream translation regulation. HER2_uORF prediction network is **Figure 4A**.

Hitoshi Endo etc. identified two SEPS encoded by uORFs of MKKS transcript with alternative polyadenylation sites at the 5′-UTR (Akimoto et al., 2013). These two SEPs are translated *in vivo* and imported onto the mitochondrial membrane. The aim of the study was to elucidate the mitochondrial localization of MKKS_uORFs and it was demonstrated that peptides encoded by uORFs were functional *in vivo*. FSPP also identified two small peptides with a length of 48 (ENST00000347364.3_182_326) and 50 (ENST00000347364.3_391_541) respectively. The peptides were translated by MKKS transcripts and the later with predicted functions (**Figure 4C**). The functions of SEP partly involve positive regulation of protein insertion into mitochondrial membrane, regulation of mitochondrial membrane potential, positive regulation of protein targeting to membrane and etc. The other functions mainly include negative regulation of transcription from RNA polymerase II promoter, negative regulation of protein binding, negative regulation of gene expression and etc. Notably, these results are in concordance with the annotated small peptides MKKS_uORF functions.

In 2010, Thomas Hamilton etc. discovered that a small peptide from the upstream smORF could mediate stress-sensitive regulation of IFRD1 mRNA decay in humans (Zhao et al., 2010). From these downloaded 38 human samples, a 52-length peptide was also found translated by an upstream region of ENST00000489994 (IFRD1). Therefore, FSPP successfully acquired significant GO annotations and two of the predicted functions are related to the stress response. These results are consistent with Professor Hamilton’s reports. In addition, two functions are respectively negative regulation of protein complex assembly and negative regulation of protein binding. It also agrees with the existing findings (**Figure 4D**).

## Discussion

The integration of overlapped results of ribosome profile sequencing (Ribo-seq) data and mass spectrometry data has predicted high-quality of SEPs. Next, both of the larger protein-coding and SEPs expression profiles were calculated in the transcription and translation level. FSPP investigates the significant correlations among the molecules and builds a relation network. At last, SEPs functions are annotated by their neighbors in the network.

FSPP takes advantage of SEPs expression features and provides a method to predict SEPs with significant functions based on the expression correlation network among the transcribed and translation level. This tool aims to the regulation of SEPs functions directly or through the interaction with other molecules. However, it appears that SEPs also play a role in the traffic of molecules across membranes and in the antimicrobial process independently. Thus, it requires the definition of new features of FSPP such as charge bias and the establishment of secondary structures to predict the transport function. On the other hand, the SEP filtering criterion on the overlap of ribosome and MS data is a little strict. As a result, there must be SEPs escaping our prediction as the false negative items. SEP expression is sensitive to the condition. So the expression matrix is comparatively sparse. In the next version of FSPP, SEP charge bias, secondary features, new identification standard and correlation computing methods can be added to acquire more functions.

All supplementary data files related to this article are available online at https://www.bioinfo.org/FSPP/example. The source code can also be downloaded from (Big Data Center Members, 2018). The website is http://bigd.big.ac.cn/biocode/tools/BT007071.

## Author Contributions

HL put forward the idea and realized the main part of the project. LX helped with the computing algorithms. LZ supplied sequencing materials and assisted with SEP expression computing. JW and BW optimized the parameter of function prediction. NS and YZ pushed the project and proposed the verification of FSPP.

## Conflict of Interest Statement

The authors declare that the research was conducted in the absence of any commercial or financial relationships that could be construed as a potential conflict of interest.

## Supplementary Material

TABLE S1 Names of 38 samples and 5 cell lines.

TABLE S2 2663 peptides predicted.

TABLE S3 Test set1.

TABLE S4 Test set2.

TABLE S5 Test set3.
